# Early Markers of Cardiac and Skeletal Muscle Metabolic Derangement in the Apc(min/+) Male Mouse

**DOI:** 10.1002/cnr2.70290

**Published:** 2025-09-23

**Authors:** Traci L. Parry, Nicole Wood, Jacob Garritson, Michael J. Muehlbauer, Louisa Tichy, Jason T. Brantley, James R. Bain, Reid Hayward

**Affiliations:** ^1^ Department of Kinesiology University of North Carolina Greensboro Greensboro North Carolina USA; ^2^ Department of Kinesiology, Nutrition, and Dietetics, and the University of Northern Colorado Cancer Rehabilitation Institute University of Northern Colorado Greeley Colorado USA; ^3^ Sarah W. Stedman Nutrition and Metabolism Center, Duke Molecular Physiology Institute Duke University School of Medicine Durham North Carolina USA; ^4^ Division of Endocrinology, Metabolism, and Nutrition, Department of Medicine Duke University School of Medicine Durham North Carolina USA

**Keywords:** atrophy, cachexia, cancer, cardiac, heart, muscle, tumor

## Abstract

**Background and Aims:**

Cancer cachexia is a metabolic and wasting disease that occurs in up to 80% of cancer patients. Currently, there are no clear diagnostic criteria, its effects are irreversible, and it cannot be treated. Most patients progress undetected to late stages of cancer cachexia, stop responding to traditional treatment, and die without an effective intervention. While the literature has begun to characterize late (refractory) cachexia muscle metabolic changes, less is known about early changes that may precede obvious muscle dysfunction and wasting. Therefore, this investigation aimed to characterize early phase heart and skeletal muscle metabolic changes in a preclinical model of colorectal cancer.

**Methods:**

The Apc(min/+) mouse spontaneously forms tumors along the intestinal tract and is a well‐accepted preclinical colorectal cancer model. To identify early changes in muscle metabolism during colorectal cancer development, heart and gastrocnemius tissues from 15‐week‐old male Apc(min/+) and litter‐matched non‐carrier mice (wildtype) were analyzed by untargeted GC/MS metabolomics.

**Results:**

In the heart, metabolic pathways related to taurine/hypotaurine metabolism; biosynthesis of unsaturated fatty acids; alanine, glutamate, and aspartate; arginine and proline; and arginine biosynthesis were affected by colorectal cancer. In skeletal muscle, metabolic pathways involving arginine biosynthesis; alanine, glutamate, aspartate, and proline metabolism were affected by cancer cachexia. Taken together, these data demonstrate altered arginine metabolism and proline metabolism in hearts and skeletal muscle of cachectic mice. Interestingly, cardiac muscle showed a non‐preferential fuel switch towards less energetically favorable glycolysis (vs. fatty acid metabolism) that coincided with cardiac dysfunction, while skeletal muscle exhibited glucose dysregulation and possible insulin resistance.

**Conclusion:**

These data characterize early cardiac and skeletal muscle metabolic derangements that lead to muscle dysfunction and atrophy during colorectal cancer. Such data could help identify patients in early phases of cachexia or identification of cardiac and skeletal muscle specific therapeutic targets aimed at early intervention.

## 1 Introduction

Cancer remains one of the leading causes of death worldwide. In the US, approximately 1 in 4 deaths are due to cancer [1]. According to the American Cancer Society, taken as a whole, gastrointestinal (GI) cancers have the highest incidence and are the second leading cause of death. Adenomatous polyposis coli (*APC*) is widely known as a tumor suppressor, with mutations in this gene found in ~80% of human colorectal cancers [2], as well as in many pancreatic and gastric cancers [3, 4]. Cancer cachexia, a muscle and fat wasting disorder, occurs at the highest rate in colorectal cancers (85%) and pancreatic cancers (83%) [5, 6]. Cancer cachexia is understudied and uncured, largely because there are neither clear diagnostic criteria nor standard treatment strategies, leading many to believe that it contributes to up to 80% of deaths in cancer patients [7, 8, 9]. During cachexia, patients experience a catabolic state as the body uses fat and muscle for energy, resulting in a severe wasting syndrome. This process cannot be fully reversed and leads to progressive and permanent impairment and poor quality of life [10, 11, 12, 13, 14, 15]. Most critically, cachectic cancer patients become non‐responsive to traditional cancer treatments, severely reducing survival rates [16]. As a result, there is a need to better understand the early phases of cancer cachexia that precede the more obvious physical characteristics, like muscle and fat wasting and severe weight loss that are hallmarks of late phase refractory cachexia. Such information could be instrumental in creating early‐ or mid‐phase diagnostic criteria for identifying patients currently developing cachexia, or in the identification of early‐mid phase therapeutic targets aimed at slowing, stopping, or reversing cancer cachexia development.

Since cachexia appears to be metabolically driven, high‐throughput tools such as metabolomics are well positioned to interrogate physiologic changes that occur during tumor growth and cancer cachexia. It is likely that cachexia involves changes in complex biochemical pathways involving interactions among multiple tissues. Previous investigations have shown altered lipid homeostasis, impaired glucose metabolism, and elevated amino acid metabolism in skeletal muscle during the late phase (refractory) cancer cachexia [17, 18, 19, 20, 21, 22]. Only a handful of studies have targeted earlier timepoints in the cancer cachexia continuum, identifying alterations in the metabolism of amino acids and lipoproteins as factors contributing to skeletal muscle wasting [21, 23, 24, 25]. Unfortunately, the majority of this data has been collected from in vitro models or models where tumors grow rapidly and thus do not follow a clinically relevant tumor growth curve and timeline. This highlights a need for investigations of earlier timepoints and in clinically relevant models where tumor formation and growth—and resulting muscle wasting—follow a more gradual trajectory as is observed in human cancers [26, 27]. Such data could strengthen the identification of early phase diagnostic criteria or potential therapeutic targets aimed at metabolic changes in the progression of cancer cachexia.

While there has been significant focus placed on metabolic changes in skeletal muscle, such changes in the heart are often overlooked [28, 29]—particularly metabolic changes that occur during early‐mid phase cancer cachexia and how this may ultimately impact cardiac function. Thus, it is critical to investigate multiple muscle types in order to gain a more holistic understanding of early cardiac and skeletal muscle metabolic changes and how they may contribute to muscle function decline and cancer cachexia development. Such data could shed light on muscle‐specific metabolic markers of the development of cancer cachexia, informing a more targeted therapeutic approach to treatment.

Since cachexia occurs at a high rate in colorectal cancers [5, 6] and at a disproportionately high rate in males [30, 31], this study utilized the preclinical male Apc(min/+) colorectal cancer mouse model [32] to investigate metabolic changes across different muscle tissues. In the preclinical Apc(min/+) mouse model, a min (multiple intestinal neoplasia) mutation in the *APC* gene causes mice to form neoplasia throughout the intestines beginning around 4 weeks of age, progressing to cachexia by 14 weeks of age, and finally to severe cachexia by 20–24 weeks of age [33, 34, 35]. Spontaneous formation of intestinal tumors in these APC‐mutant mice closely resembles key metabolic, inflammatory, and physiological aspects of human cancers [30, 33, 36, 37, 38]. Therefore, the present study aims to identify early changes in cardiac and skeletal muscle metabolism during colorectal cancer development in the preclinical male Apc(min/+) mouse model.

## 2 Methods

### 2.1 Study Design and Apc(min/+) Mouse Model

The Apc(min/+) mouse, a preclinical intestinal cancer model capable of inducing cancer cachexia [33, 34], was utilized to help identify key early phase cachexia metabolite changes in the heart and skeletal muscle. Male Apc(min/+) (*n* = 6) and litter‐matched (sibling and age‐matched) wildtype (WT, *n* = 7) mice were purchased from Jackson Laboratory (strain #: 002020; Maine, USA) and maintained at the University of Northern Colorado Animal Research Facility. All mice were bred onsite in a temperature‐controlled animal facility with a 12:12 light–dark cycle. During the length of the protocol, mice were housed in standard mouse cages and provided with distilled water and standard rodent chow ad libitum. All studies were approved by the University of Northern Colorado's Institutional Animal Care and Use Committee. All mice were carefully monitored throughout the study (starting at 8 weeks of age) for general health, body conditioning [39], and food and water intake. Since the purpose of this study was to determine metabolic changes that might occur during the early phases of cancer cachexia, 15 weeks of age was chosen as the endpoint based on previous studies indicating mice progress to “cachexia” (i.e., early phase cachexia) by 14 weeks of age and progress to late phase “refractory” cachexia by 20–24 weeks of age [33, 34, 35]. As such, 15‐week‐old male mice were assessed via echocardiography, euthanized by pentobarbital overdose followed by cervical crush, and tissues were collected and flash frozen in liquid nitrogen.

### 2.2 Echocardiography

In vivo cardiac function was analyzed at 15 weeks of age using echocardiography, as previously described by our laboratory [40]. Transthoracic echocardiography (GE Vivid 7 Dimension) M‐mode images of the left ventricle (LV) were obtained by short axis view to measure LV‐end systolic and diastolic diameters and fractional shortening. Echocardiography flow images were performed via apical view using pulsed wave Doppler and used to measure maximal flow velocity through the mitral valve (LV filling time) and aortic valve (LV ejection time). For all echocardiographic measures, data were averaged from three consecutive cardiac cycles. All M‐mode and Doppler measurements were made in accordance with guidelines established by the American Society of Echocardiography and were analyzed using the UltraLinq ultrasound management system (New York, NY).

### 2.3 Untargeted GC–MS Metabolomics

Untargeted gas chromatography–mass spectrophotometry (GC–MS) metabolomics was used to characterize the metabolome in each tissue. All tissues were prepared for untargeted metabolomics according to Banerjee et al. [41]. Tissues were pulverized by mortar and pestle on dry ice, then homogenized in ice cold buffer (50% acetonitrile, 50% water, and 0.3% formic acid) at a standard concentration of 25 mg/475 μL, and frozen at −80°C. Next, samples were deproteinated by methanol precipitation and spiked with an internal standard for retention‐time, and metabolites were made volatile. GC–MS generally followed the methods of Roessner et al. [42] and electron ionization was used to generate positive ions at 70 eV, scanned from 600 to 50 m/z. Raw data were processed, and peaks were annotated as metabolites using a previously established retention time‐locked spectral library of metabolites, which is built upon the Golm Metabolome Library, the Fiehn GC/MS Metabolomics RTL Library, and other spectral libraries. Peaks were cross‐referenced and confirmed by SpectConnect.

### 2.4 Statistical Analysis

All data are presented as mean ± standard deviation (SD). All statistics were performed using the statistical software GraphPad Prism (La Jolla, CA, USA) or MetaboAnalyst (v5.0). For body masses, muscle metrics, and echocardiography, a Student's *T*‐test was performed for each variable to determine differences between groups. All analyses were two‐tailed, and an alpha level of 0.05 was used to define statistical significance. For metabolomics analyses, each tissue's data (heart, gastrocnemius) was log transformed and Pareto scaled [41, 43]. Using MetaboAnalyst (v5.0), an unsupervised Principal Component Analysis (PCA) was performed to determine the principal source of variance, and a Partial Least Squares Discriminant Analysis (PLS‐DA) was performed to determine the discrimination between variables [44, 45, 46, 47]. Cross validation was conducted to calculate R2 (coefficient of determination) and Q2 (cross‐validated R2). Variable Importance in Projection (VIP) analyses were conducted to determine which metabolites contributed most significantly to differences between groups, with VIP scores > 2 considered significant [48]. A Student's *T*‐test was performed to determine significant differences between groups. All analyses were two‐tailed, and a False Discovery Ratio (FDR) corrected alpha level of 0.05 was used to define statistical significance. Significant metabolites (identified as *t*‐test significant and FDR corrected *p* < 0.05 or VIP > 2) were matched to metabolomics pathways using the Pathway Analysis feature and Pathway Enrichment feature in MetaboAnalyst to determine relationships among significant metabolites. Heat maps of the metabolite data were generated and Euclidean sorted in MetaboAnalyst (v5.0).

## 3 Results

### 3.1 Body and Muscle Masses

Body and muscle masses of wildtype (WT) and Apc(min/+) mice are displayed in Table 1. No significant differences existed between groups for body mass at the start of the study. By the end of the study, Apc(min/+) mice exhibited tumors along the intestinal tract and had significantly lower body mass compared to litter‐matched WT mice, consistent with other studies of Apc/min mice of similar age [33, 34, 35]. There was no significant difference in gastrocnemius mass or gastrocnemius mass normalized to final body mass between Apc(min/+) mice and litter‐matched WT mice. Although muscle atrophy is a common feature of late phase (refractory) cancer cachexia, a small number of studies have shown that changes at the molecular level (e.g., metabolic changes) precede obvious muscle wasting, atrophy, and dysfunction. In fact, preclinical cancer models have shown that metabolic, inflammatory, and other signaling alterations may precede physical muscle wasting [21, 23, 24, 25, 33, 49]. Since the mice in the study are 15 weeks old at the time of sacrifice, it is possible that the wasting phenotype is not yet present despite metabolic changes in the muscle. Therefore, the timing of our model—investigating possible muscle metabolic changes that occur prior to severe muscle wasting (that occur at later stages of cachexia)—appears to appropriate.

**TABLE 1:** Body mass and muscle metrics in wildtype and Apc(min/+) mice.

Metric	Wildtype	Apc(min/+)
Initial body mass (g)	23.6 ± 0.35	23.3 ± 0.41
Final body mass (g)	23.1 ± 0.75	21.6 ± 0.93*
Gastrocnemius (g)	0.144 ± 0.008	0.143 ± 0.012
Gastrocnemius/final body mass (mg/g)	0.63 ± 0.04	0.66 ± 0.04

*Note:* Units for each variable are denoted in parentheses. Values are expressed as Mean ± SD; analyzed by Student's *t*‐test. Wildtype, *N* = 7; Apc(min/+), *N* = 6.

* *p* < 0.01.

### 3.2 Cardiac Structure and Function

**FIGURE 1:**
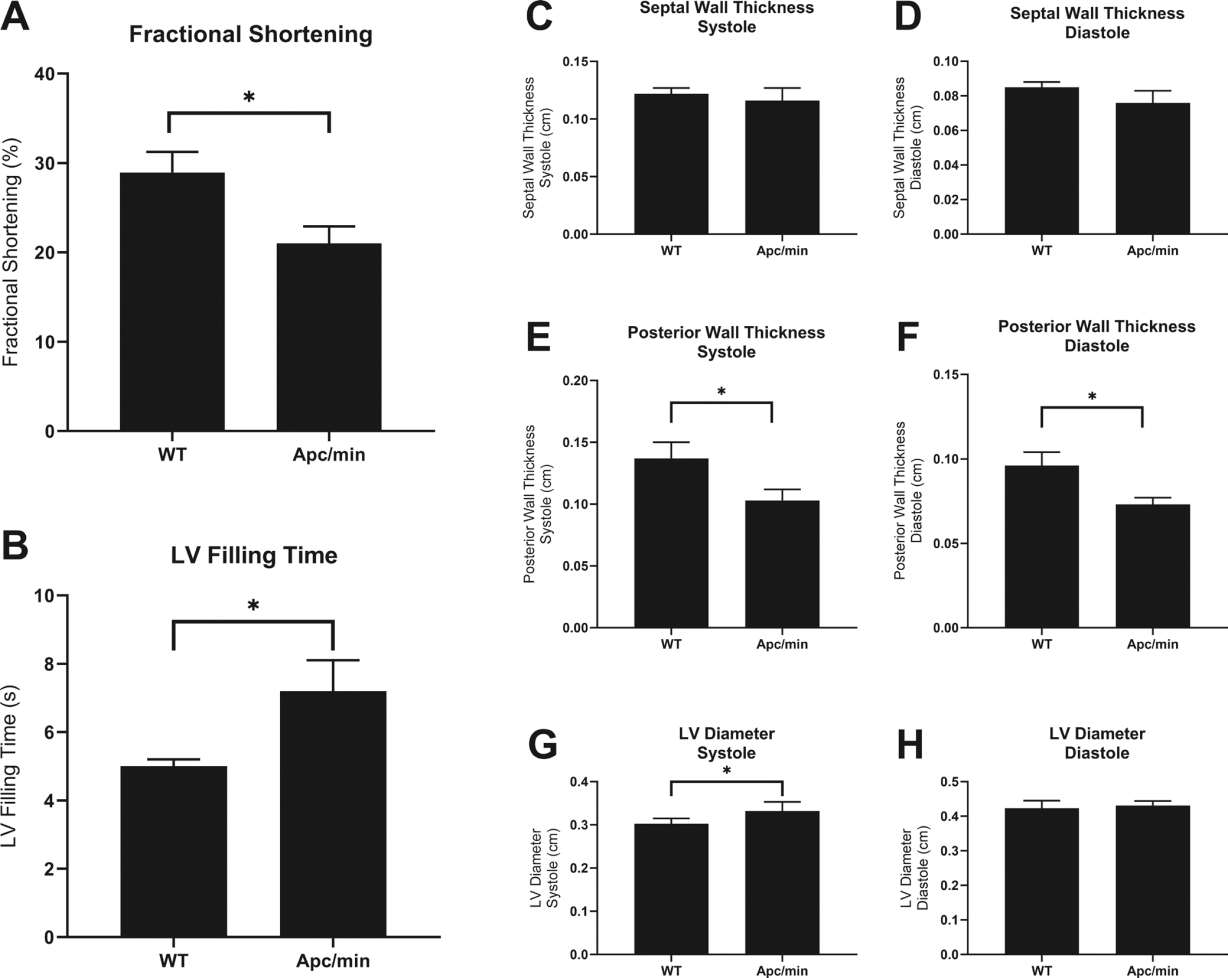
Echocardiography reveals cardiac dysfunction in Apc(min/+) mice. (A) Fractional shortening is decreased in Apc(min/+) mice. (B) Left ventricular filling time is increased in Apc(min/+) mice. No changes were observed in septal wall thickness during systole (C) or diastole (D). Posterior wall thickness during systole (E) and diastole (F) are decreased in Apc(min/+) mice. Left ventricular diameter was increased in Apc/min mice during systole (G) but not during diastole (H). LV = left ventricular. Values are expressed as Mean ± SD; analyzed by Student's *t*‐test. Wildtype (WT), *N* = 7; Apc(min/+), *N* = 6. *p < 0.05.

Echocardiography revealed that 15‐week‐old Apc(min/+) male mice exhibit significantly worse cardiac function compared to litter‐matched sibling WT controls. Apc(min/+) mice exhibited decreased fractional shortening and slower left‐ventricular filling time (Figure 1). This coincides with significant (*p* < 0.05) thinning of the posterior wall during systole and diastole and thinning of the septal wall during diastole (*p* < 0.05) (Figure 1). These data indicate that by 15 weeks of age, Apc(min/+) mice are beginning to develop a cardiac muscle wasting phenotype indicative of dilated cardiomyopathy. Interestingly, these data indicate that cardiac wasting may precede skeletal muscle wasting during the development of cachexia.

### 3.3 Cardiac Muscle Metabolism

**FIGURE 2:**
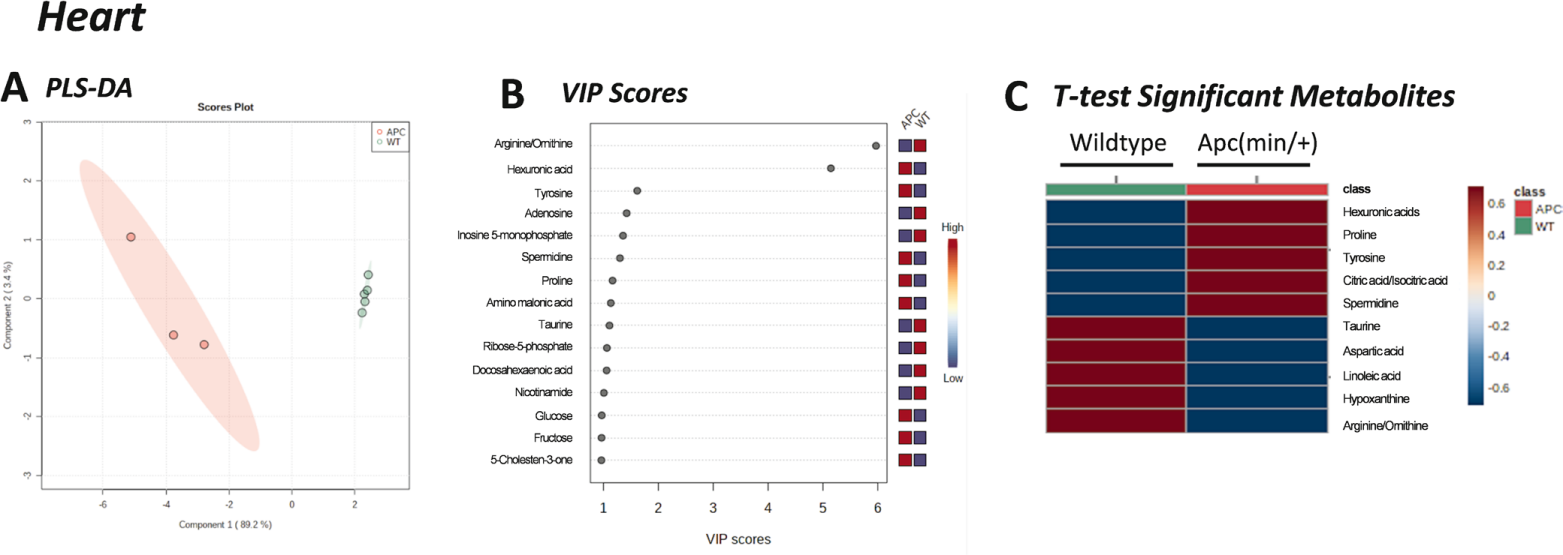
Untargeted metabolomics analysis of the heart in Apc(min/+) mice. (A) Partial Least Squares Discriminant Analysis (PLS‐DA) plot. (B) Variable Importance in Projection (VIP) Scores (considered statistically significant if VIP > 2). (C) Heart *T*‐test significant metabolites (*p* < 0.05). Red = increased/high; Blue = decreased/low. Collectively, 10 features were identified as significantly altered in the cardiac muscle in Apc(min/+) compared to wildtype mice. APC, Apc(min/+) mice (*N* = 3); WT, wildtype mice (*N* = 5).

**FIGURE 3:**
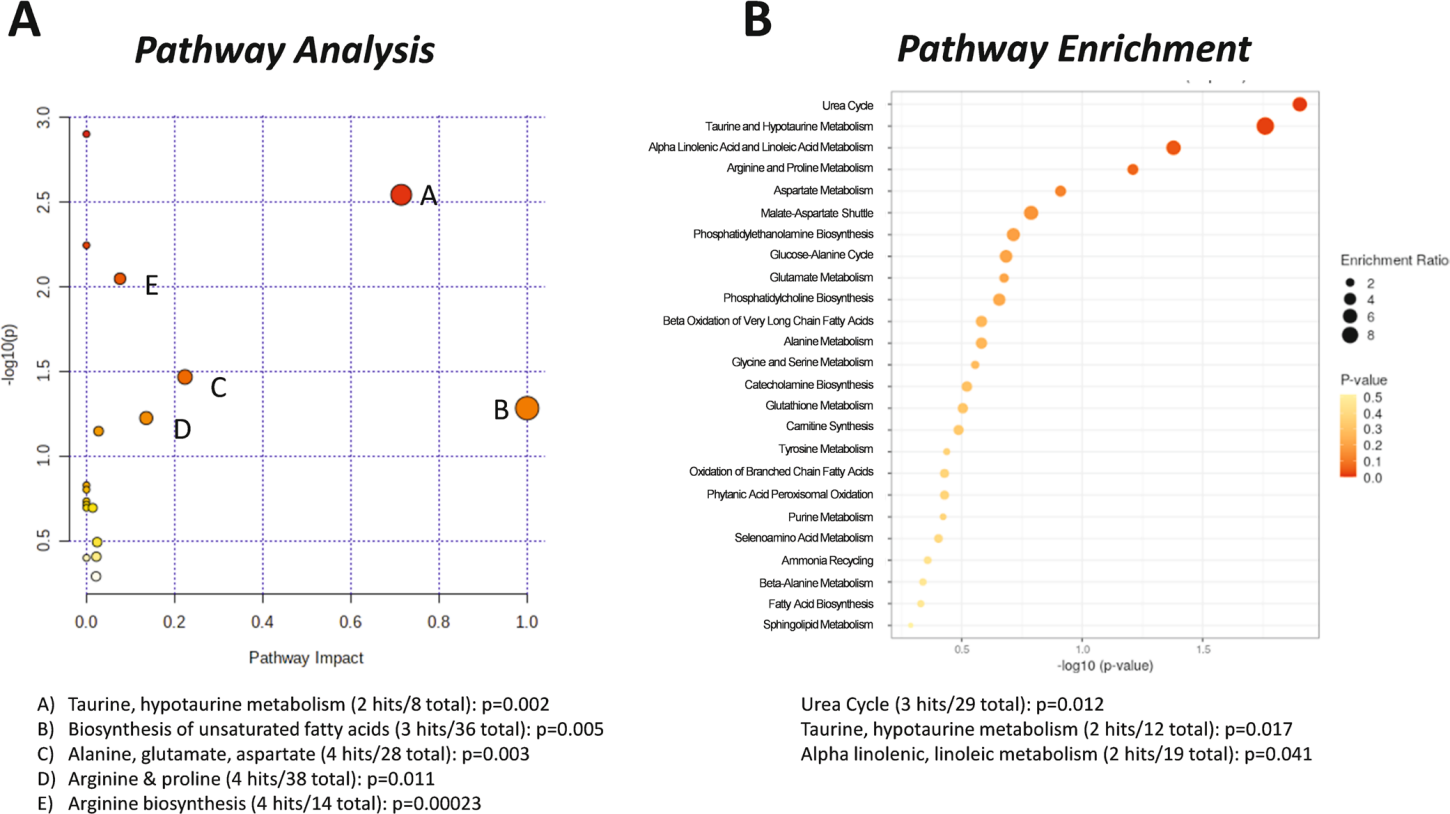
Pathway analysis of the heart in Apc(min/+) mice. Ten metabolites were identified as significantly different in the Apc(min/+) hearts compared to wildtype controls (FDR corrected *p* < 0.05, VIP > 2). (A) Untargeted metabolomics pathway analysis of cardiac significantly altered metabolites using the KEGG database. (B) Pathway enrichment analysis performed on metabolites ranked by significance and impact on pathway. Larger dots indicate more hits per metabolic pathway (enrichment). Darker red dots indicate greater statistical significance.

Untargeted metabolomics of the heart muscle queried 137 metabolites. Partial least squares discriminant analysis (PLS‐DA, Figure 2A) was used to determine discriminating features for the separation between groups. A clear separation between groups was observed, with the first two components explaining 89.2% and 3.4% of the total variance, respectively. Cross validation was conducted, yielding R2: 0.998 and Q2: 0.939, indicating a good model. In the heart, five metabolites were significantly increased in Apc(min/+) mice compared to litter‐matched WT mice: hexuronic acids, proline, citric acid, spermidine, and tyrosine; and five metabolites were significantly decreased: taurine, hypotaurine, aspartic acid, arginine, and linoleic acid (Figure 2C). These data coincide with the variable importance in projection (VIP) scores (Figure 2B), where arginine was significantly decreased and hexuronic acid was significantly increased in the hearts of Apc(min/+) mice compared to litter‐matched controls. Ten features were identified as significantly different by *t*‐test (FDR corrected *p* < 0.05) and VIP (VIP > 2) analyses: hexuronic acids, proline, citric acid, spermidine, tyrosine, taurine, hypotaurine, aspartic acid, arginine, and linoleic acid. Pathway analysis (Figure 3A) of these significantly different cardiac metabolites revealed pathways involved in taurine/hypotaurine metabolism; biosynthesis of unsaturated fatty acids; alanine, glutamate, and aspartate; arginine and proline; and arginine biosynthesis. Enrichment analysis (Figure 3B), based on these same significantly altered metabolites, showed pathways involved in the urea cycle, taurine/hypotaurine metabolism, and alpha linolenic and linoleic metabolism. These data suggest that the heart, a highly aerobic organ dense with mitochondria, was catabolizing amino acids and low levels of fatty acids in the Apc(min/+) mice, indicating that the heart is likely stressed during colorectal cancer and may be shifting away from the more energetically favorable aerobic metabolism of glucose.

### 3.4 Skeletal Muscle Metabolism

**FIGURE 4:**
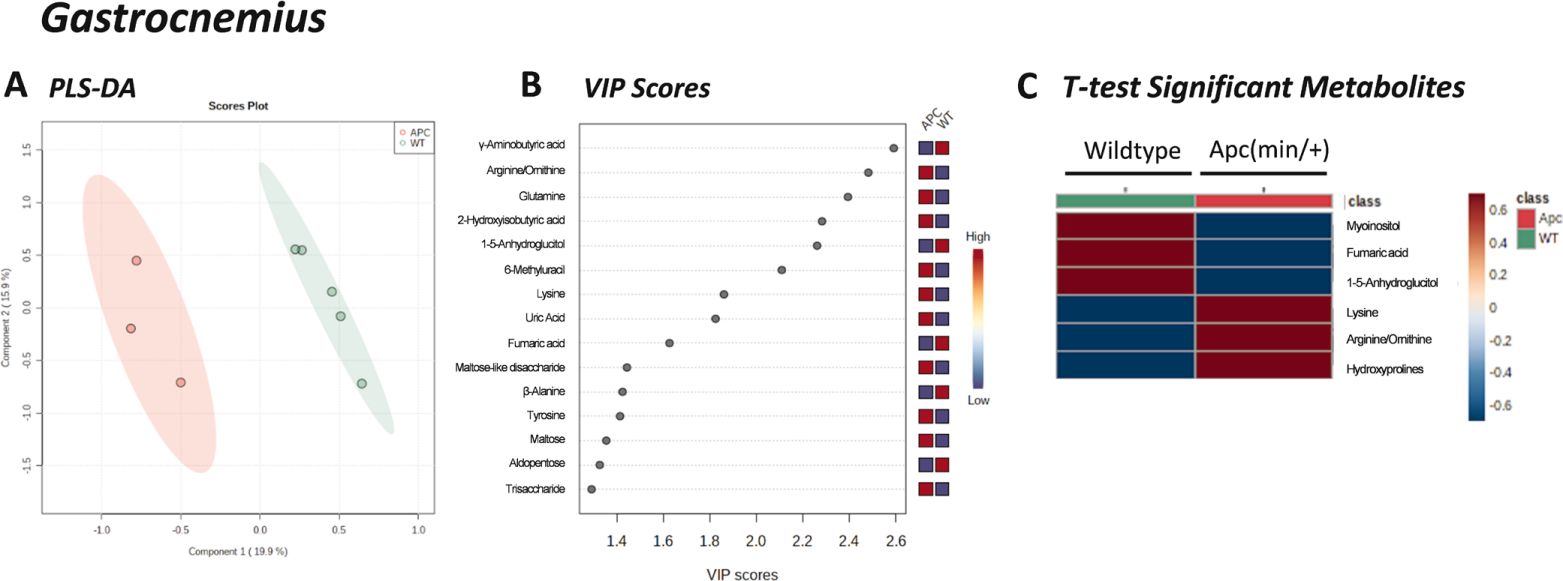
Untargeted metabolomics analysis of the mixed fiber gastrocnemius skeletal muscle in Apc(min/+) mice. (A) Partial Least Squares Discriminant Analysis (PLS‐DA) plot. (B) Variable Importance in Projection (VIP) Scores (considered statistically significant if VIP > 2). (C) Mixed‐fiber gastrocnemius *T*‐test significant metabolites (*p* < 0.05). Red = increased/high; Blue = decreased/low. Collectively, nine features were identified as significantly altered in the gastrocnemius muscle in Apc(min/+) compared to wildtype mice. APC, Apc(min/+) mice (*N* = 3); WT, wildtype mice (*N* = 5).

**FIGURE 5:**
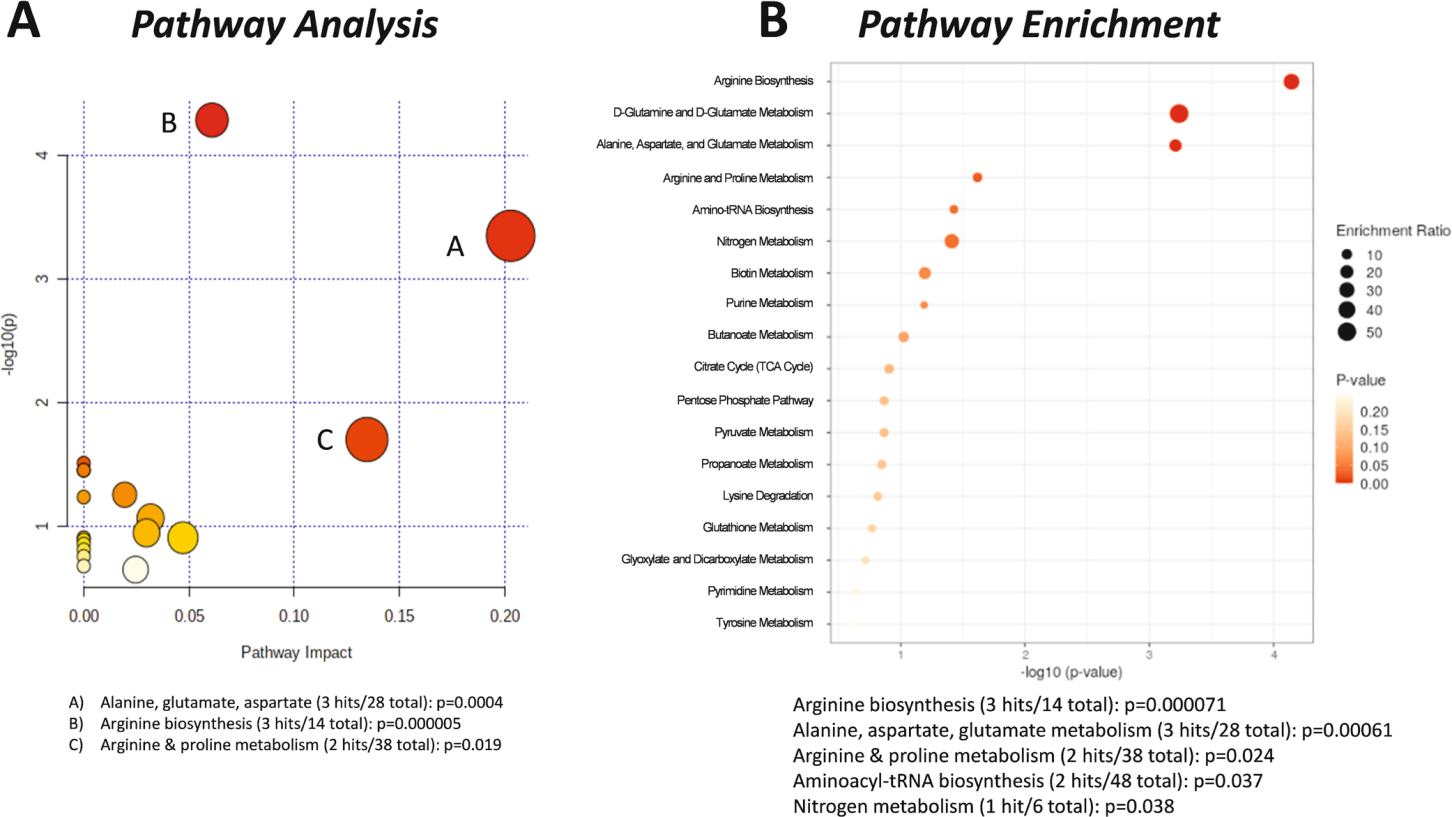
Pathway analysis of the mixed fiber gastrocnemius skeletal muscle in Apc(min/+) mice. Nine features were identified as significantly different in the Apc(min/+) gastrocnemius compared to wildtype controls (FDR corrected *p* < 0.05, VIP > 2). (A) Untargeted metabolomics pathway analysis of gastrocnemius significantly altered metabolites using the KEGG database. (B) Pathway enrichment analysis performed on metabolites ranked by significance and impact on pathway. Larger dots indicate more hits per metabolic pathway (enrichment). Darker red dots indicate greater statistical significance.

Untargeted metabolomics of the mixed‐fiber gastrocnemius skeletal muscle reported on 135 metabolites. PLS‐DA distinguished features for the separation between groups (Figure 4A). A clear separation between tumor‐bearing and wild‐type mice was observed, with the first two components explaining 19.9% and 15.9% of the total variance, respectively. Cross validation was conducted, yielding R2: 0.987 and Q2: 0.643, indicating a moderately good model. In the gastrocnemius, three metabolites were significantly increased in Apc(min/+) mice compared to litter‐matched WT mice: lysine, ornithine/arginine, and hydroxyproline; and three metabolites were significantly decreased: myoinositol, 1‐5‐anhydroglucitol, and fumaric acid (Figure 4C). These data correlate with the variable importance in projection (VIP) scores (Figure 4B), which additionally identified glutamine and 2‐hydroxyisobutyric acid as metabolites significantly increased in the Apc(min/+) mice, and *gamma*‐aminobutyric acid significantly decreased in Apc(min/+) mice compared to litter‐matched WT mice. Nine features were identified as significantly different by *t*‐test (FDR corrected *p* < 0.05) and VIP (VIP > 2) analyses: lysine, ornithine/arginine, hydroxyproline, myoinositol, 1‐5‐anhydroglucitol, fumaric acid, glutamine, 2‐hydroxyisobutyric, and *gamma*‐aminobutyric acid. Pathway analysis (Figure 5A) of these significantly different gastrocnemius skeletal muscle metabolites revealed pathways involved in alanine, glutamate, and aspartate; arginine and proline; and arginine biosynthesis. Enrichment analysis (Figure 5B), based on these same significantly altered metabolites, showed pathways involved in arginine biosynthesis; alanine, aspartate, glutamate metabolism; arginine and proline metabolism; aminoacyl‐tRNA biosynthesis; and nitrogen metabolism. These data suggest the mixed‐fiber skeletal muscle, which is normally reliant on glucose oxidation for energy, is stressed during colorectal cancer due to impaired glucose metabolism.

## 4 Discussion

Cancer cachexia, a complex multifactorial wasting disorder, is highly prevalent in colorectal, gastric, and pancreatic cancer survivors [6]. Unfortunately, cancer cachexia is poorly understood. There are no clear diagnostic criteria or treatment strategies. The *APC* mutation is found in ~80% of human colorectal cancers [2], as well as pancreatic and gastric cancers [3, 4]. This study sought to identify key metabolite changes in heart and skeletal muscle in Apc(min/+) mice, a preclinical intestinal cancer model. In this model, a point mutation of the *APC* gene causes mice to develop multiple intestinal neoplasia beginning around 4 weeks of age, with cachexia onset by 14 weeks of age, and severe cachexia by 20–24 weeks of age [33, 34, 35]. Spontaneous formation of intestinal tumors in these APC‐mutant mice closely resembles key metabolic, inflammatory, and physiological aspects of human cancers [30, 33, 36, 37, 38]. While there is an overall lack of information regarding physiological changes that occur during cachexia, metabolic changes occurring in skeletal muscle during the late phase (refractory) cachexia have been given the most focus. As such, metabolic changes that occur during the earlier phases of cancer cachexia ‐ as well as those occurring in the heart ‐ remain understudied and poorly understood. Therefore, the goal of this work was to shed light on how colorectal cancer may elicit *early* phase changes in the metabolic profile of the heart and skeletal muscle to help inform possible early diagnostic criteria and muscle‐specific therapeutic targets.

In this study, heart and mixed‐fiber skeletal muscles (gastrocnemius) of 15‐week old male Apc(min/+) (tumor‐bearing) mice and non‐tumor‐bearing wildtype (WT) mice were investigated for key metabolite changes by untargeted metabolomics. Tumor‐bearing male Apc(min/+) mice exhibited thinning of left ventricular walls, slowed ejection time, and depressed fractional shortening compared to WT littermates. These data indicate that cardiac muscle atrophy, likely due to the stress of colorectal cancer, contributed to cardiac dysfunction and a failing heart phenotype. This is in step with recent findings that cancer survivors can exhibit significant cardiac atrophy, thinned ventricular walls, and reduced ventricular mass [29, 50]. Moreover, survivors of advanced forms of cancer, likely experiencing late‐stage refractory cachexia, exhibit severely depressed cardiac function that significantly complicates treatment strategies and clinical outcomes [50, 51]. It is interesting that in the present study, cardiac atrophy appears to precede skeletal muscle wasting, and it is possible that a failing heart contributes to skeletal muscle wasting via poor perfusion of skeletal muscle tissue. Therefore, monitoring cancer patients for ventricular dysfunction and cardiac atrophy may be a prudent strategy to identify patients in the early stages of cancer cachexia development.

During cancer‐mediated cardiac dysfunction and atrophy, there are key signaling changes at play. A key aim of the present study was to determine potential early cardiac metabolic changes that occur during colorectal cancer cachexia. Our investigation of 15‐week‐old Apc(min/+) male mice reveals altered amino acid, glucose, and fatty acid metabolism in the myocardium—which aligns with metabolic signatures found in serum from human cancer cachexia patients [52]. In our model, the observed metabolic disruption coincided with thinning of the left ventricular posterior wall, reduced fractional shortening, and slowed left ventricular filling time. This data is in accordance with Manne et al. [53] that found 12‐week‐old Apc(min/+) mice exhibited smaller hearts compared to WT controls and this coincided with increased AMPK and AKT activation and enhanced beclin1 protein expression. In this same study, older 20‐week‐old Apc(min/+) continued to decline, exhibiting a stronger phenotype with further diminished protein synthesis rates, metabolic dysfunction, and upregulated protein degradation [53]. They surmised that inflammatory signaling (e.g., IL‐6) was likely to play a role in the observed cardiac wasting phenotype. Interestingly, in skeletal muscle, proinflammatory TLR9 coordinates with beclin1 to activate AMPK under energetic stress [54], which may lead to metabolic dysfunction and catabolic signaling as the heart attempts to maintain metabolic homeostasis under stress. In a male C26 tumor model comparing different cancer cachexia timepoints, cardiac mass was lowered by 8% at day 15 and by 21% at day 27, and this coincided with progressively increased serum pro‐inflammatory cytokines [31]. Since tumors are known to release inflammatory factors [55], it is possible that systemic inflammation is the key trigger initiating tumor‐mediated cardiac metabolic dysfunction and myocardial wasting.

There appears to be a critical relationship between an upregulation of cardiac inflammation and a progressively worsening imbalance in protein metabolism, in which catabolism exceeds anabolism [31, 53, 56, 57, 58]. Key mediators of this imbalance are likely the ubiquitin‐proteasome system and the autophagy lysosome system. Increased spermidine levels are associated with heart failure during transaortic constriction [59]. Interestingly, spermidine—which is elevated in the hearts of our tumor‐bearing mice—has been shown to increase autophagy in mammalian cells [60]. Furthermore, an upregulation of autophagy markers beclin‐1, p62, and LC3B‐II has been observed in cachectic rodent models and in the hearts of cancer survivors [29, 53, 58, 61]. Therefore, excessive levels of autophagy, initiated by spermidine, may be one mechanism for early‐phase cardiac wasting and subsequent cardiac dysfunction observed in our tumor model and in cancer cachectic patients.

The heart is a highly aerobic organ, with high mitochondrial density and reliance on fatty acid metabolism. Interestingly, tumor‐secreted factors appear to alter transcription pathways in cardiomyocytes. An in vitro mouse cardiomyocyte study showed fatty acid metabolism genes (related to storage, transport, and oxidation) were most dysregulated when exposed to C26 murine tumor cell conditioned media [62]. Additionally, cachectic C26 tumor‐bearing mouse hearts showed decreased PPARα, decreased CPT1β, an *alpha*‐ to *beta*‐ myosin heavy chain isoform switch (i.e., adult to fetal), and a switch in glucose transporters from GLUT4 (adult) to GLUT1 (fetal), indicating a decreased reliance on fatty acids and increased reliance on less energetically favorable glycolytic metabolism. This coincided with a significant decline in cardiac function that included depressed left ventricular ejection fraction and thinning of the interventricular wall [63]. Together, this could help explain the observed perturbations of fatty acid biosynthesis and linoleic acid metabolism and the resulting cardiac dysfunction during colorectal cancer cachexia in the present study.

Taurine has been shown to be cardioprotective during coronary artery disease [64], which may be related to its anti‐inflammatory effects [65]. Similarly, arginine was shown to attenuate cardiac muscle loss during chronic heart failure [66] and to help improve recovery following major cardiac events [67]. Interestingly, in our tumor‐bearing (Apc(min/+)) mice, taurine and arginine were decreased in the heart. This may indicate that cardioprotective mechanisms are compromised during colorectal cancer cachexia, demonstrating that tumor presence can significantly impact cardiac metabolism, structure, and function.

While weight loss and reduced food intake are long‐standing factors used to identify patients at risk for cachexia‐related adverse outcomes, a “systemic inflammatory response” has more recently moved to the forefront as a defining characteristic of cancer cachexia. This systemic inflammatory response has wide‐ranging negative effects on the host, and one specific consequence is a reduction in lean body mass. During cachexia, severe skeletal muscle wasting leads to loss of lean mass, frailty, fatigue, and reduced quality of life and survival [68]. Since, by time of diagnosis, cancer cachexia often progresses to late stages, where muscle and body wasting are apparent and patients become untreatable, a key aim of the present study was to determine potential skeletal muscle metabolic changes that occur early during colorectal cancer cachexia. Our investigation in 15‐week‐old male Apc(min/+) mice showed altered pathways involved in amino acid metabolism (arginine, proline, alanine, aspartate, glutamate, and glutamate), aminoacyl‐tRNA biosynthesis, and nitrogen metabolism—which agrees with metabolic signatures found in serum from human cancer cachexia patients [52]. Interestingly, metabolic perturbations were observed prior to evidence of significant muscle wasting, indicating that metabolic changes may precede and thus trigger signaling pathways that lead to protein degradation and muscle wasting. This data is in accordance with an investigation of time course changes in Apc(min/+) mice, indicating that as early as 13 weeks, Apc(min/+) mice showed detectable increases in metabolic, inflammatory, and muscle wasting genes (*fkbp5, 4ebp1, cebpd, foxo1, foxo3, bnip3, lcb3, mafbx*) in the quadriceps and this continued to progress and worsen in 23 weeks old Apc(min/+) mice [69]. In a time course study of C26 tumor bearing mice, O'Connell et al. [23] showed that amino acids were among the first metabolites to show alterations, beginning at just 4 days of C26 tumor implant—prior to evidence of muscle wasting or discernable tumor growth. Over the course of the study, serum amino acids (including ornithine and proline) were altered during early phases of tumor bearing, while metabolites involved in fatty acid oxidation were altered in later phases of tumor bearing and this occurred with progressively larger tumors and muscle wasting [23]. In a study comparing early phase (9 days of Walker‐256 tumor‐bearing) and late phase (12–14 days of Walker‐256 tumor‐bearing), early phase was associated with altered amino acid metabolism, aminoacyl‐tRNA biosynthesis, and nitrogen metabolism—including matching decrease in myoinositol and decrease in body mass despite no significant difference in gastrocnemius muscle mass [21]. In this study, Walker‐256 tumor‐bearing mice continued to progress to a cancer cachexia phenotype, with clear muscle wasting and altered amino acid and fatty acid metabolism that correlated with larger tumors [21]. This early phase cachexia data also agrees with human serum signatures from pre‐cachexia (early phase cachexia) patients, showing increased myoinositol and decreased hydroxyproline [70]. Therefore, it is possible that inflammation give rise to altered amino acid metabolism—including myoinositol, hydroxyproline, proline, and ornithine—which could be indicators of early phase cancer cachexia muscle wasting, while changes in fatty acid oxidation and amino acids could demarcate later phase cancer cachexia.

Cardiac dysfunction, whether from cardiotoxic cancer treatments or from the cancer itself, has the potential to exacerbate wasting at the level of the skeletal muscle due to poor perfusion that directly impacts its metabolic and functional properties. The combination of poor perfusion and the systemic inflammatory response causes progressive degradation of skeletal muscle proteins to amino acids during cancer cachexia. In a compound mutant mouse tumor model (pVillin‐KRAS^V12G^ × Apc^1638N^), lysine, proline, and arginine were significantly increased in skeletal muscle during cancer cachexia, and this was associated with a negative energy charge, reduced ATP levels, and mitochondrial dysfunction [71]. Elevated levels of prolines and hydroxyprolines (hyperprolinemia) could cause derangement in systemic glucose metabolism via amino acid toxicity, leading to insulin dysregulation from impaired *beta*‐cell function [72]. Others have reported elevated hydroxyproline levels in a mouse model of hindlimb unloading that coincided with muscle atrophy and weakness [73]. C26 tumor‐bearing mice showed elevated levels of serum hydroxyproline levels that correlated with skeletal muscle collagen turnover and thus implicated in skeletal muscle remodeling [74]. Similarly, proline and lysine were elevated in the gastrocnemius of aged rats, and this was associated with elevated markers of protein degradation (MuRF1, Atrogin1), an elevated marker of inflammation (activated NF‐kB), and reduced muscle mass [75]. Finally, ornithine, an amino acid that participates in the urea cycle, was upregulated in elderly males with muscle loss, which coincided with muscle weakness [76]. In an Apc(min/+) model of cancer cachexia, liver gluconeogenic‐related genes were upregulated in 13 weeks old and 23 weeks old Apc(min/+) mice, indicating the liver's role in glucose production via gluconeogenesis [69]. Therefore, the breakdown of muscle structural proteins and increase in amino acids appears to be associated with muscle weakness and metabolic perturbations.

The mixed‐fiber gastrocnemius muscle is highly glycolytic. Recently, 1,5‐anhydroglucitol was shown to be a marker of glycemic control. In patients with type 2 diabetes, this marker was shown to be negatively correlated with hemoglobin A1C and fasting glucose levels [77]. In our tumor model, 1,5‐anhydroglucitol was decreased in the gastrocnemius of the Apc(min/+) mice, which may indicate disrupted glucose homeostasis and recent excursions into hyperglycemia and hyperglycosuria. In line with this is the finding of decreased myoinositol in the gastrocnemius of Apc(min/+) mice, which has been implicated in insulin sensitivity and resistance [78]. During impaired glucose‐homeostasis states such as diabetes, skeletal muscle can become insulin resistant, resulting in reduced glucose uptake (elevated blood glucose). However, treatment with myoinositol (via injection or via diet) improved insulin sensitivity in skeletal muscles of female mice while also maintaining muscle mass [78]. This may be due to myoinositol's ability to increase GLUT4 translocation to the membrane in skeletal muscle to lower plasma glucose and insulin levels [79]. Finally, treatment with arginine resulted in improved insulin sensitivity in type 2 diabetes patients [80], indicating ties between arginine metabolism and insulin signaling. Our data support the notion that during the development of cancer cachexia, glycolytic skeletal muscles may become insulin resistant, causing metabolic dysregulation that can lead to eventual muscle weakness and wasting.

While not investigated in this study, mitochondrial dysfunction is also apparent during cancer cachexia development, is likely triggered by proinflammatory cytokines, and contributes to skeletal muscle dysfunction and eventual wasting [81]. In mice, mitochondrial degeneration was apparent by just 2 weeks after Lewis lung carcinoma (LLC) tumor implant, and this preceded evidence of skeletal muscle atrophy [25]. Similarly, in weight stable Apc(min/+) mice, mitochondrial mRNA for *Pgc‐1α*, *Pparα*, and *Mfn2* were significantly decreased in quadriceps, indicating that mitochondrial biogenesis is impacted prior to obvious body and muscle wasting in the development of cancer cachexia [25]. Furthermore, in LLC tumor‐bearing male mice, cachectic skeletal muscle showed increased lipid content (myosteatosis) and decreased lipid droplet‐mitochondrial contact, which contributed to impaired lipid metabolism [82]. Similarly, factors secreted from breast cancer cells appear to cause in vitro skeletal muscle cells to undergo myosteatosis, leading to impaired mitochondrial function [83]. Therefore, markers of mitochondrial dysfunction may be additional markers for diagnosis of early phase versus late phase cancer cachexia or skeletal muscle specific therapeutic targets.

Cancer cachexia remains unresolved and uncured. In this study, we identify muscle‐specific early phase metabolic alterations during cancer cachexia. In the heart, a shift towards the less energetically favorable glycolytic metabolism occurs, possibly due to mitochondrial dysfunction, indicative of a failing heart. In the mixed‐fiber gastrocnemius, insulin resistance and dysregulated glycolytic metabolism are a possible consequence of early phase cancer cachexia. Interestingly, we observed thinning of ventricle walls and cardiac dysfunction prior to skeletal muscle wasting, despite detecting changes in metabolism in both muscle types. Therefore, monitoring cardiac function over the cancer continuum may help identify patients at risk of or currently developing cancer cachexia. In both tissues, there is evidence of altered amino acid metabolism, likely driven by inflammatory cytokines from the tumor. Understanding crosstalk among the tumor, the heart, skeletal muscle, and other organs remains essential to understanding the pathophysiology of cancer cachexia to identify early diagnostic criteria as well as muscle‐specific treatment targets and strategies. Multi‐timepoint and multi‐tissue studies are critically needed to pinpoint mechanisms and discover therapeutic targets.

## Author Contributions

Conceptualization: T.L.P. Methodology: T.L.P., J.R.B., M.J.M., and R.H. Investigation, resources, analysis: T.L.P., N.W., J.G., M.J.M., L.T., J.T.B., J.R.B., and R.H. Writing – original draft: T.L.P. and R.H. Writing – reviewing and editing: T.L.P., L.T., M.J.M., J.R.B., and R.H. Project administration, supervision: T.L.P. Funding acquisition: T.L.P., J.R.B., M.J.M., and R.H.

## Conflicts of Interest

The authors declare no conflicts of interest.

## Data Availability

The data that support the findings of this study are available from the corresponding author upon reasonable request.
